# Hygienic Handwriting

**Published:** 1887-08

**Authors:** 


### Hygienic Handwriting.

Schubert, of Neuremberg, has made a careful study of various kinds of script, and the bearing which the use of each has upon the hygiene of the eyes among children, and the following are his conclusions :

As a practical conclusion to be drawn from his observations, Schubert lays down the rule that all children should be taught a perpendicular handwriting. Even if the erect median position of copy book be not actually better than the oblique median, still the teacher cannot tell when inspecting writing done at home what absurd position may have been adopted in writing it, if the child is permitted to write anything but perpendicular letters. These letters can only be executed in the erect median position. It may be possible for adults to write more rapidly a slanting than a perpendicular hand, but children are not required to write rapidly, but in a manner that does not tend to deform their vertebral columns or their eyes. In many countries now-a-days, and in times past, perpendicular handwriting alone obtains, and Schubert appends a series of fac similes of German handwriting in every century from the 8th to the 18th inclusive. From this it is seen that slanting letters were not adopted to any extent until the 17th century.—*Ophthalmic Review.*

